# Deep Learning Network with Spatial Attention Module for Detecting Acute Bilirubin Encephalopathy in Newborns Based on Multimodal MRI

**DOI:** 10.3390/diagnostics13091577

**Published:** 2023-04-28

**Authors:** Huan Zhang, Yi Zhuang, Shunren Xia, Haoxiang Jiang

**Affiliations:** 1 Key Laboratory of Biomedical Engineering of Ministry of Education, Zhejiang University, Hangzhou 310027, China; 2 Department of Radiology, Affiliated Children’s Hospital of Jiangnan University, Wuxi 214036, China

**Keywords:** residual network, spatial attention module, acute bilirubin encephalopathy, newborn

## Abstract

Background: Acute bilirubin encephalopathy (ABE) is a significant cause of neonatal mortality and disability. Early detection and treatment of ABE can prevent the further development of ABE and its long-term complications. Due to the limited classification ability of single-modal magnetic resonance imaging (MRI), this study aimed to validate the classification performance of a new deep learning model based on multimodal MRI images. Additionally, the study evaluated the effect of a spatial attention module (SAM) on improving the model’s diagnostic performance in distinguishing ABE. Methods: This study enrolled a total of 97 neonates diagnosed with ABE and 80 neonates diagnosed with hyperbilirubinemia (HB, non-ABE). Each patient underwent three types of multimodal imaging, which included T1-weighted imaging (T1WI), T2-weighted imaging (T2WI), and an apparent diffusion coefficient (ADC) map. A multimodal MRI classification model based on the ResNet18 network with spatial attention modules was built to distinguish ABE from non-ABE. All combinations of the three types of images were used as inputs to test the model’s classification performance, and we also analyzed the prediction performance of models with SAMs through comparative experiments. Results: The results indicated that the diagnostic performance of the multimodal image combination was better than any single-modal image, and the combination of T1WI and T2WI achieved the best classification performance (accuracy = 0.808 ± 0.069, area under the curve = 0.808 ± 0.057). The ADC images performed the worst among the three modalities’ images. Adding spatial attention modules significantly improved the model’s classification performance. Conclusion: Our experiment showed that a multimodal image classification network with spatial attention modules significantly improved the accuracy of ABE classification.

## 1. Introduction

Neonatal jaundice, also known as neonatal hyperbilirubinemia, is a prevalent condition in newborns, identified by yellowing of the skin and whites of the eyes caused by the accumulation of bilirubin in the bloodstream. Bilirubin, a yellow pigment formed during the breakdown of red blood cells, is regulated by the liver among healthy individuals; however, in newborns, the liver may not fully develop, leading to the accumulation of bilirubin in the bloodstream, resulting in jaundice. Although neonatal jaundice is generally harmless and resolves itself within a few weeks, increased levels of bilirubin could cross the blood–brain barrier, leading to the death of brain cells, subsequently causing acute bilirubin encephalopathy (ABE) [1,2]. If left untreated, ABE can progress to a severe condition known as kernicterus, leading to possible neurological damage or even death. Thus, monitoring newborns with ABE and seeking medical attention if symptoms persist or worsen are crucial.

In clinical settings, the total serum bilirubin concentration (TSB) serves as a typical approach to evaluate neonatal jaundice. A physician’s diagnosis of acute bilirubin encephalopathy is primarily based on the results of laboratory biochemical tests and clinical characteristics. Although a TSB level exceeding 340 μmol/L is believed to be the critical threshold for the development of serum bilirubin encephalopathy [3], the free fraction of bilirubin in the blood is the most neurotoxic component, and the TSB concentration cannot be used to measure the bilirubin level in the brain directly. Consequently, it is an imperfect indicator of the risk of brain damage from bilirubin. Additionally, collecting blood through skin puncture can be traumatic for newborns, exposing them to the danger of infection [4]. Hence, discovering a non-invasive way to detect brain changes caused by bilirubin, an essential element in the diagnosis of ABE, is necessary.

Magnetic resonance imaging (MRI) is a powerful diagnostic and therapeutic tool for neurological diseases, including bilirubin encephalopathy [5]. It provides detailed images of the brain, enabling physicians to make accurate diagnoses and formulate effective treatment plans for their patients. Multimodal MRI including T1-weighted, T2-weighted, and diffusion-weighted images, which involves the acquisition of multiple images using different types of MRI sequences, has become increasingly popular in medical imaging analysis. The combination of these modalities can improve diagnostic accuracy by providing complementary information about tissue properties.

Several studies investigated the T1, T2, and ADC images of patients with bilirubin encephalopathy, finding that these imaging methods are beneficial in diagnosing and producing additional information that might enhance the diagnostic accuracy [6]. Hyperintense signals in the bilateral pallidum in T1-weighted images are typical of the acute phase of bilirubin encephalopathy, representing characteristic features [7]. This outcome could occur due to the high level of neural activity in the basal ganglia, including the globus pallidus (GP), leaving them more vulnerable to the effects of bilirubin accumulation, and the resulting damage to these regions can be detected via MRI as T1 hyperintensity; however, this radiological signature was found to not be applicable in all cases through further research. It was discovered that some non-ABE patients with high levels of bilirubin also exhibited a high signal intensity in the GP, making it even more difficult to differentiate between ABE and non-ABE using only T1-weighted images. Meanwhile, abnormal T2-weighted imaging findings are rare during the acute phase; however, in severe or progressive cases of the illness, hyperintense T2 signals may appear in the bilateral pallidum. These T2 signals coincide with the T1 hyperintensity concerns in the acute phase. Limited studies have examined the clinical application of diffusion-weighted imaging (DWI) in the diagnosis of ABE [8]; however, investigators discovered that the ADC values from DWI are highly correlated with the bilirubin levels in the bloodstream. Neurological assessment and imaging techniques, such as magnetic resonance spectroscopy (MRS) and DWI, can also help distinguish ABE from other illnesses that produce similar symptoms [9,10]. Currently, methods based on traditional machine learning and deep learning are widely used in medical image analysis and the diagnosis of clinical diseases, achieving tremendous success [11,12]. Liu et al. initially used machine learning methods to distinguish ABE from normal myelination by manually segmenting the region of interest (ROI), extracting features, and selecting features based on T1-weighted images [13]. Wu et al. employed the deep learning networks ResNet18 and DenseNet201 for the classification of multimodal MRI images. The results from their study showed that multimodal images improved the ABE classification performance [12]. Although these experiments achieved a good performance in classification, they had certain limitations and issues. For example, the machine learning methods required manual segmentation of the GP and defined corresponding grayscale and texture features, followed by further feature selection using a two-sample *t*-test. These processes involved too much human intervention and were not conducive to automated processing and future clinical use. The multimodal deep learning network simply concatenated T1, T2, and ADC data into a 3D input for the model. This approach shared the same network weights across different modalities, which was not ideal for multimodal data and hindered the network’s ability to learn distinct features from each modality.

The spatial attention module in a convolutional neural network (CNN) is designed to selectively focus on certain regions within an image while downplaying or ignoring others [14]. This module can improve the performance of the CNN by allowing it to better recognize and classify objects within an image. The spatial attention module works by using a set of weights to assign importance values to different parts of the input image. These weights are learned during training and are based on the features that are most relevant to the task at hand. By adjusting these weights, the CNN can focus its attention more closely on the key areas of an image, such as the face of a person or the lesion area of the brain.

Multimodal MRI-based deep learning models have emerged as a promising approach for medical image analysis due to their ability to integrate information from multiple modalities. Recent works in this area have achieved remarkable success in various applications, including the detection of abnormalities and diseases in brain imaging. Zhang et al. proposed a deep-learning-based method for the automated detection of enlarged perivascular spaces (EPVS) in brain MRI images [15]. The proposed model was trained on a large dataset of MRI images and achieved a high accuracy in detecting EPVS, outperforming other existing methods. Another related work by Guo et al. proposed a method for glioma subtype classification using multiple MRI modalities and a decision fusion strategy to improve accuracy [16]. These works on multimodal MRI-based deep learning models have demonstrated promising results in medical image analysis, particularly in the detection, classification, and segmentation of brain abnormalities and diseases.

Therefore, in this study, we created a multimodal MRI image classification network based on ResNet18 that can differentiate ABE from the non-ABE control group (high bilirubin, HB). For each modality of data, we used the respective ResNet18 as the backbone to extract the features and then concatenated the features before the fully connected layer. Additionally, we introduced spatial attention modules into the ResNet18 network blocks to further enhance the classification performance of the model. In addition, we investigated the influence of different combinations of modalities on the classification results.

## 2. Materials and Methods

### 2.1. Study Subjects

The data were collected during routine clinical examinations at the Affiliated Children’s Hospital of Jiangnan University in 2020–2022, and all research protocols were approved by the Clinical Research Ethics Committee. We recruited 177 newborns with high bilirubin levels for this study, of which 97 were diagnosed with ABE and 80 were diagnosed with non-ABE. Experienced pediatricians confirmed the diagnosis of all subjects based on the clinical examination results and the bilirubin-induced neurologic dysfunction (BIND) score, which is a scale used to evaluate the severity of ABE. The scores range from 1 to 9, with 1–3 indicating mild, 4–6 indicating moderate, and 7–9 indicating severe ABE [17]. Neonates without ABE did not have the corresponding clinical neurological symptoms.

### 2.2. MRI Acquisition

We collected all MRI images using a 1.5 T Siemens MRI scanner according to experimental requirements. The T1-weighted images were acquired using the following parameters: TR/TE, 200/4.8 ms; slice thickness, 4 mm, 20 slices; flip angle, 90°; matrix size, 272 × 288; field of view, 217 × 230 mm. The T2-weighted images were acquired using the following parameters: TR/TE, 2800/98 ms; slice thickness, 4 mm, 20 slices; flip angle, 150°; matrix size, 256 × 256; field of view, 230 × 230 mm. The diffusion-weighted images were acquired using the following parameters: TR/TE, 3800/95 ms; slice thickness, 4 mm, 20 slices; flip angle, 90°; matrix size, 164 × 168; field of view, 224 × 230 mm; b value, 1000 s/mm². All images underwent manual inspection by pediatricians to ensure that the image quality met the requirements for subsequent data analysis.

### 2.3. Image Pre-Processing

**Figure 1:**
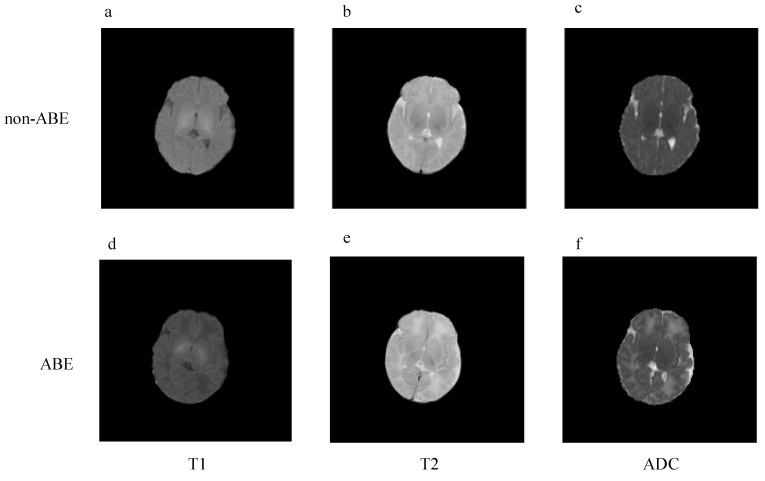
Axial images of three modalities of MRI: (**a**–**c**) represent T1, T2, and ADC images of non-ABE neonates, respectively; (**d**–**f**) represent T1, T2, and ADC images of ABE neonates, respectively.

We applied image pre-processing in the following steps: (1) skull stripping (FSL v7.0: SynthStrip, https://fsl.fmrib.ox.ac.uk/fsl/fslwiki/, accessed on 15 January 2023) [18,19], and (2) normalizing the image intensity to a range of 0–1 and resizing the image to 224 × 224 (Figure 1). To improve the computational efficiency, we selected four contiguous slices around the GP from each modality of the T1, T2, and ADC images as input for the models. We performed all pre-processing steps using Python and FSL with Ubuntu20.0.

### 2.4. Deep Learning Framework and Spatial Attention Module

**Figure 2:**
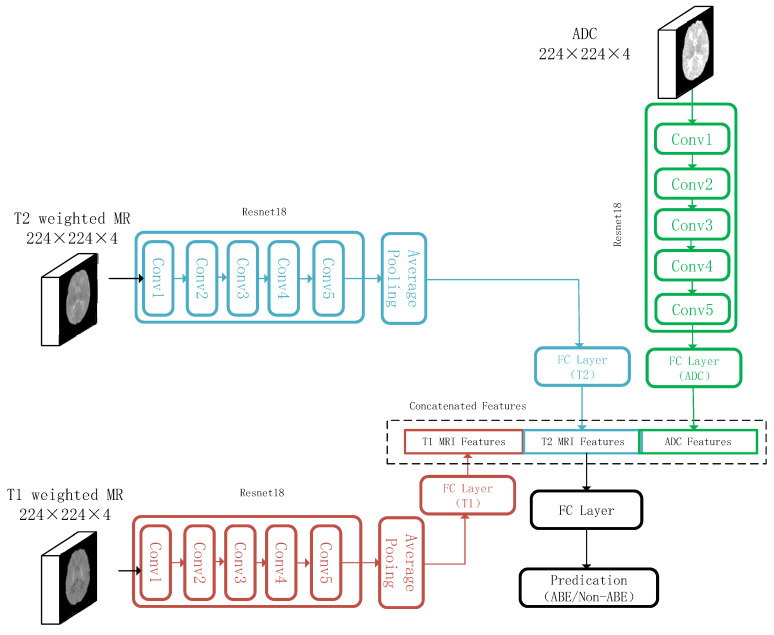
Deep learning architectures for the multimodal ABE prediction model. The feature extractors for T1-weighted images, T2-weighted images, and ADC images were used in ResNet18. MRI input images were selected to include four consecutive layers where the GP was located, and they were cropped to a size of 224 × 224 × 4. The output of the feature extractors for each modality was concatenated, and a fully connected layer of the prediction for ABE was used.

We used ResNet18 as the backbone to build a multimodal image classification network (Figure 2), where ResNet18 was used for image feature extraction [20]. Subsequently, we fused the multimodal features and constructed a fully connected layer to distinguish between ABE and non-ABE patients. We used transfer learning methods to initialize the model parameters effectively and improve the training performance. To counteract the issues of limited training subjects and overfitting during the training process, we used data augmentation methods, including randomly translating images horizontally and vertically by −60 to 60 pixels, rotating images by −60 to +60 degrees, and scaling images 0.8 to 1.2 times.

**Figure 3:**
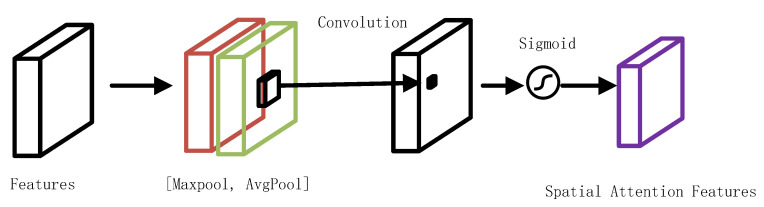
Diagram of the spatial attention module. The spatial attention module separately calculated the maximum pooling output and the average pooling output along the channel axis. These results were then concatenated and passed through a convolutional layer and sigmoid activation function to obtain the spatial attention features.

**Figure 4:**
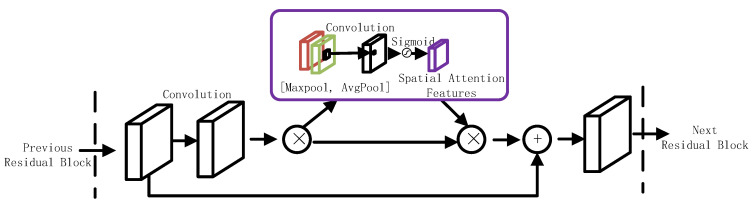
Diagram of the spatial attention module integrated with a residual block in ResNet18. The diagram shows the specific connection position of the spatial attention module in the residual block of ResNet18.

In this paper, we introduced spatial attention modules (SAMs) into the residual network blocks and analyzed the effect of the spatial attention modules on the model classification performance. The detailed structure of the attention modules and their integration with ResNet18 are illustrated in Figure 3 and Figure 4, respectively [14].

### 2.5. Model Evaluation

Five-fold cross-validation was used to evaluate the generalization ability of the model, and various metrics such as the classification accuracy, the area under the curve (AUC), sensitivity, specificity, recall, and F1 score were used to evaluate the model’s classification performance. The performance metrics are presented as the mean ± standard deviation from the five-fold cross-validation.

To verify the classification performance of different combinations of modal images, the experiment mainly employed the following strategies: (1) single-modality data, using T1, T2, and ADC separately as model inputs; (2) dual-modality data, using T1 + T2, T1 + ADC, and T2 + ADC separately as model inputs; (3) triple-modality data, using T1 + T2 + ADC as the model input. For the multimodality inputs, the model first extracted features from each modality separately and then fused the features before finally conducting classification. To evaluate the effect of the SAMs on improving the classification performance, we conducted comparative experiments separately with models that have SAMs and models that do not have SAMs.

ImageNet-based pre-trained weight files were downloaded from the PyTorch website (https://download.pytorch.org/models/resnet18-5c106cde.pth, accessed on 13 January 2023) and used to initialize the weights of the feature extraction module in the model [21]. Training-related hyperparameters were set as follows: initial learning rate of 0.0001, maximum iteration of 140, and minibatch size of 64. The Adam algorithm was used for model training [22]. The experiment was developed with Windows 11 using Python 3.10.

## 3. Results

Table 1 shows the demographic and clinical characteristics of the patients enrolled in this study, including their gender, weight, and age. Differences in the gender distribution between groups were evaluated using the chi-square test; the result showed that there were no significant differences between the ABE and non-ABE groups (*p* = 0.15 > 0.05). As the other clinical features did not meet the assumption of normality based on the Kolmogorov–Smirnov test, we utilized the nonparametric Mann–Whitney test to evaluate differences between groups. Significant differences in age were found between the ABE and non-ABE groups, with a *p*-value of less than 0.05.

Table 2 and Table 3, respectively, demonstrate the performance of ResNet18 networks without spatial attention modules and ResNet18 networks with spatial attention modules to distinguish between ABE and non-ABE using single-modality and multimodality MRI data. The model without a spatial attention module had a classification accuracy of 0.666, 0.745, and 0.583 on T1, T2, and ADC images, respectively. The model with a spatial attention module achieved a classification accuracy of 0.674, 0.768, and 0.576 on T1, T2, and ADC images, respectively. From the results of the single-modality experiment, it was observed that the T2-weighted images achieved the best classification performance in both models with and without attention modules. However, the ADC images did not perform as well and showed the lowest classification accuracy and AUC in comparison. By comparing the implementation results with and without spatial attention modules, it was found that spatial attention modules helped to improve the overall classification performance of the model, especially on T2 images.

In the multimodality experiment, the combination of T1 and T2 achieved the best classification accuracy, area under the curve, sensitivity, and F1 score in both models with and without attention modules. Among the dual-modality data, the classification accuracy for T1 + ADC and T2 + ADC was found to be lower than that of their corresponding single modalities, indicating that the ADC images did not contribute to improving the model’s classification performance. The overall classification performance of models with attention modules was better than that of those without attention modules, particularly in terms of enhancing the specificity.

**Figure 5:**
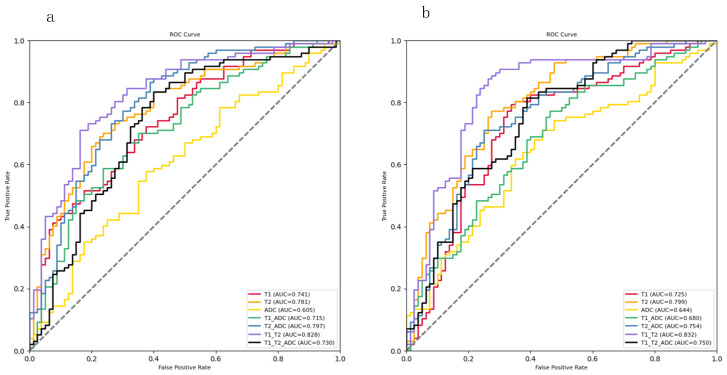
ROC curves for distinguishing ABE from non-ABE using different modal MRI features. The ROC curves were obtained from testing single-modality data T1, T2, and ADC, and multimodality data T1 + ADC, T2 + ADC, T1 + T2, and T1 + T2 + ADC using ResNet18 with and without SAMs. (**a**) ROC curves based on ResNet18 without SAMs. (**b**) ROC curves based on ResNet18 with SAMs.

Figure 5 shows the ROC curves of the model tests with different combinations of MRI data. The results indicated that increasing the number of multimodal images helped to improve the AUC of the model. The combination of T1 and T2 achieved the best AUC in both ResNet18 with and without SAMs (with SAMs, 0.832; without SAMs, 0.828). Compared to the combination of other imaging modalities, it was found that the combination of ADC images with other modalities was not conducive to improving the AUC of the model. In fact, it led to a decrease in the model’s classification performance.

## 4. Discussion

In this study, we proposed a deep learning network based on multimodal MRI images to distinguish between ABE and non-ABE. We validated whether multimodal images had superior ABE diagnosis performance compared to single-modality images and compared the classification performance of ResNet18 models with spatial attention modules and without spatial attention modules. Our experimental results showed that multimodal image fusion improved the ABE prediction compared to single-modality T1-weighted, T2-weighted, and ADC images, and the inclusion of a spatial attention module helped to improve the overall classification performance of the model, particularly in terms of specificity.

The results of the single-modality image experiments showed that T2-weighted images had the best classification performance, followed by T1-weighted and ADC images. The ADC images performed the worst among the three modalities’ images, which was also confirmed in the multimodality image experiments. This finding may be because ADC images did not show significant differences between the ABE and non-ABE groups in our dataset [23].

Acute bilirubin encephalopathy is typically characterized by a high symmetric signal intensity in T1-weighted images in the GP, subthalamic nucleus (SN), and hippocampus regions [7]; however, in chronic bilirubin encephalopathy, the high signal intensity in T2-weighted images is more pronounced in the GP and SN compared to T1-weighted images [24,25]. Our experiment’s results showed that the classification performance of the T2-weighted images was better than that of the T1-weighted images. Furthermore, among all combinations of modal images, the T1- and T2-weighted image combination achieved the best classification accuracy, AUC, sensitivity, and specificity.

Spatial attention modules have been shown to be effective in improving the performance of various models in image classification and object recognition [14]. We introduced spatial attention modules into residual blocks and adjusted their weights through training, so that the model could focus its attention on key areas of the image, such as the high-signal area of the pallidum. Our results showed that SAMs improved the overall classification performance of the model, especially in terms of specificity, compared to the control experiment.

Despite the promising results obtained in this study, there are some limitations that need to be addressed in future research. One of the main limitations is the sample size, which was relatively small and drawn from a single source. In order to increase the generalizability of the model, future studies should include larger and more diverse samples from multiple sources. Another limitation of our study is that the 2D-ResNet model design did not fully utilize the 3D information available in the images. To address this limitation, future research could consider using more advanced models such as 3D convolutional neural networks (3D-CNNs) that can effectively capture the spatial information in volumetric data. Additionally, incorporating other modalities of MRI data such as MRS, perfusion magnetic resonance imaging, and clinical information could further improve the diagnostic accuracy of the model. This could be achieved through the use of cross-modality attention modules, which allow for the fusion of information across modalities. Finally, in order to improve the interpretability of the model and facilitate its adoption by clinicians, future research could explore the use of explainable AI techniques such as Transformers [26,27,28]. These models have been shown to be effective at generating interpretable representations of medical images and may help to improve the diagnostic capabilities of the model. Overall, these advancements in methodology hold great promise for enhancing the accuracy and clinical utility of MRI-based diagnosis of ABE.

## 5. Conclusions

In this study, we developed a network framework for multimodal MRI image classification using ResNet18 as the backbone. Our results demonstrate that the accuracy of ABE classification can be significantly improved by utilizing multimodal image combinations, particularly the T1 + T2 combination (accuracy = 0.763 ± 0.029), compared to using single-modality images. Moreover, we incorporated a spatial attention module into the residual blocks, further enhancing the classification performance, with the highest accuracy achieved using the T1 + T2 combination (accuracy = 0.808 ± 0.069). This finding suggests that a multimodal classification network with a SAM is a promising approach for the clinical diagnosis of ABE. Future research can explore the integration of more advanced MRI techniques and larger datasets to further validate the effectiveness of our approach.

## Figures and Tables

**Table 1:** The demographic and clinical characteristics of the subjects.

Clinical Features	ABE (*n* = 97)	Non-ABE (*n* = 80)	*p*-Value
Gender (male/female)	49/48	49/31	0.15
Weight (kg)	3.80 ± 0.70	4.06 ± 1.20	0.19
Age (days)	9.86 ± 5.76	16.04 ± 12.57	0.03

**Table 2:** The classification performance of ResNet18 without a spatial attention module based on single-modality and multimodality MRI data.

MRI Modality	Accuracy	AUC	Sensitivity	Specificity	Precision Score	F1 Score
T1	0.666 ± 0.107	0.706 ± 0.141	0.669 ± 0.166	0.662 ± 0.084	0.701 ± 0.088	0.681 ± 0.121
T2	0.745 ± 0.062	0.804 ± 0.071	0.708 ± 0.155	**0.787 ± 0.071**	**0.805 ± 0.030**	0.745 ± 0.088
ADC	0.583 ± 0.079	0.633 ± 0.011	0.527 ± 0.096	0.650 ± 0.011	0.648 ± 0.081	0.579 ± 0.080
T1 + ADC	0.656 ± 0.109	0.721 ± 0.088	0.662 ± 0.197	0.650 ± 0.130	0.695 ± 0.089	0.670 ± 0.124
T2 + ADC	0.690 ± 0.066	0.779 ± 0.043	0.672 ± 0.194	0.712 ± 0.144	0.747 ± 0.054	0.693 ± 0.100
T1 + T2	**0.763 ± 0.029**	**0.816 ± 0.021**	**0.836 ± 0.096**	0.675 ± 0.103	0.761 ± 0.039	**0.793 ± 0.034**
T1 + T2 + ADC	0.673 ± 0.052	0.674 ± 0.069	0.690 ± 0.118	0.650 ± 0.130	0.712 ± 0.061	0.695 ± 0.062

The bold values indicate the maximum value of each performance metric across all modal combinations.

**Table 3:** The classification performance of ResNet18 with a spatial attention module based on single-modality and multimodality MRI data.

MRI Modality	Accuracy	AUC	Sensitivity	Specificity	Precision Score	F1 Score
T1	0.674 ± 0.155	0.736 ± 0.143	0.58 ± 0.164	**0.788 ± 0.18**	0.777 ± 0.179	0.659 ± 0.156
T2	0.768 ± 0.029	0.796 ± 0.039	0.763 ± 0.149	0.775 ± 0.144	**0.82 ± 0.075**	0.778 ± 0.048
ADC	0.576 ± 0.016	0.638 ± 0.034	0.525 ± 0.081	0.637 ± 0.120	0.646 ± 0.050	0.573 ± 0.034
T1 + ADC	0.696 ± 0.115	0.713 ± 0.122	0.652 ± 0.174	0.750 ± 0.088	0.755 ± 0.087	0.694 ± 0.130
T2 + ADC	0.644 ± 0.123	0.735 ± 0.053	0.525 ± 0.211	0.787 ± 0.114	0.726 ± 0.158	0.598 ± 0.209
T1 + T2	**0.808 ± 0.069**	**0.808 ± 0.057**	**0.856 ± 0.083**	0.750 ± 0.076	0.806 ± 0.057	**0.830 ± 0.064**
T1 + T2 + ADC	0.678 ± 0.086	0.764 ± 0.090	0.650 ± 0.120	0.713 ± 0.169	0.744 ± 0.102	0.686 ± 0.085

The bold values indicate the maximum value of each performance metric across all modal combinations.

## Data Availability

The data that support the findings of this study are available on request from the corresponding author. The data are not publicly available due to privacy or ethical restrictions.
